# Evaluating the Neutralizing Ability of a CpG-Adjuvanted S-2P Subunit Vaccine Against Severe Acute Respiratory Syndrome Coronavirus 2 (SARS-CoV-2) Variants of Concern

**DOI:** 10.1093/cid/ciab711

**Published:** 2021-11-05

**Authors:** Chia-En Lien, Tsun-Yung Kuo, Yi-Jiun Lin, Wei-Cheng Lian, Meei-Yun Lin, Luke Tzu-Chi Liu, Jinyi Cheng, Yu-Chi Chou, Charles Chen

**Affiliations:** Medigen Vaccine Biologics Corporation, Taipei City, Taiwan; Institute of Public Health, National Yang-Ming Chiao Tung University, Taipei City, Taiwan; Department of Biotechnology and Animal Science, National Ilan University, Yilan County, Taiwan; Medigen Vaccine Biologics Corporation, Taipei City, Taiwan; Medigen Vaccine Biologics Corporation, Taipei City, Taiwan; Medigen Vaccine Biologics Corporation, Taipei City, Taiwan; Medigen Vaccine Biologics Corporation, Taipei City, Taiwan; Medigen Vaccine Biologics Corporation, Taipei City, Taiwan; Biomedical Translation Research Center, Academia Sinica, Taipei City, Taiwan; Medigen Vaccine Biologics Corporation, Taipei City, Taiwan; Temple University, Philadelphia, Pennsylvania, USA

**Keywords:** COVID-19, SARS-CoV-2, subunit SARS-CoV-2 vaccine, immunogenicity, variant of concern

## Abstract

**Background:**

Variants of concern (VoCs) have the potential to diminish the neutralizing capacity of antibodies elicited by vaccines. MVC-COV1901 is a severe acute respiratory syndrome coronavirus 2 (SARS-CoV-2) vaccine consisting of recombinant prefusion stabilized spike protein S-2P adjuvanted with CpG 1018 and aluminum hydroxide. We explored the effectiveness of MVC-COV1901 against the VoCs.

**Methods:**

Serum samples were taken from rats and phase 1 clinical trial human subjects immunized with a low, medium, or high dose of MVC-COV1901. The neutralizing titers of serum antibodies were assayed with pseudoviruses coated with the SARS-CoV-2 spike protein of the wild-type (WT), D614G, Alpha, or Beta variants.

**Results:**

Rats vaccinated twice with vaccine containing high doses of antigen retained high levels of neutralization activity against the Beta variant, albeit with a slight reduction compared to WT. After the third dose, neutralizing titers against the Beta variant were noticeably enhanced regardless of the amount of antigen used for immunization. In humans, vaccinated phase 1 subjects still showed appreciable neutralization abilities against the D614G, Alpha, and Beta variants, although neutralizing titers were significantly reduced against the Beta variant.

**Conclusions:**

Two doses of MVC-COV1901 were able to elicit neutralizing antibodies against SARS-CoV-2 variants with an overall tendency of inducing higher immune response at a higher dose level. The neutralizing titers to the Beta variant in rats and humans were lower than those for WT and the Alpha variant. An additional third dose in rats was able to partially compensate for the reduction in neutralization against the Beta variant. We have demonstrated that immunization with MVC-COV1901 was effective against VoCs.

## Teaser

CpG 1018–adjuvanted S-2P subunit severe acute respiratory syndrome coronavirus 2 (SARS-CoV-2) vaccine administered to rats and humans induced neutralizing antibodies against circulating SARS-CoV-2 variants of concern, including the Beta variant.

RNA viruses evolve rapidly because they can quickly accumulate mutations due to their error-prone polymerases. However, unlike most RNA viruses, coronaviruses such as severe acute respiratory syndrome coronavirus 2 (SARS-CoV-2) have large and complex RNA virus genomes with proofreading polymerases. Consequently, they mutate slowly over time, which helps to better adapt to hosts [1]. Since the beginning of the COVID-19 pandemic, mutants have been detected periodically. A number of them, variants of concern (VoCs), were found to carry mutations in the crucial receptor-binding domain (RBD), a prime target for antibody recognition and neutralization. The VoCs are designated by the World Health Organization as Alpha (B.1.1.7), Beta (B.1.351), Gamma (P.1), and Delta (B.1.617.2) variants [2–4]. The VoCs with these mutations were found to increase transmission and severity of disease or to decrease neutralization capabilities of monoclonal antibodies and vaccine-induced antibodies; as such, they pose a significant challenge on top of the existing coronavirus disease 2019 (COVID-19) global public health management [4, 5]. Major therapeutics and vaccine manufacturers such as Regeneron, Moderna, Pfizer, and AstraZeneca published reports of variants including Beta and Gamma that were highly resistant to neutralization [6–8]. Studies have attributed the resistance to antibody neutralization by Beta and Gamma variants to triple mutations K417N, E484K, and N501Y in the spike protein RBD with the E484K mutation alone, rendering the protein refractory to antibody binding via steric hindrance [6]. The industry has scrambled for strategies to combat these emerging variants, including redesigning the vaccine to elicit either variant-specific or more broadly neutralizing antibodies or administering additional vaccine doses to generate a greater immune response that compensates for the reduction in neutralization [9–11].

Medigen’s MVC-COV1901 has been shown to induce a very high level of antibody titers in preclinical studies and a phase 1 clinical trial [12, 13]. In this study, we sought to ask whether MVC-COV1901 could still confer protection against SARS-CoV-2 variants. To this end, we have examined neutralizing abilities against variants by examining antisera drawn from rats in animal studies and human clinical trial subjects vaccinated with adjuvanted S-2P.

## MATERIALS AND METHODS

### Study Overview

The study involved investigation of immunogenicity to VoCs using sera from 2 sources: rat sera from animal toxicology studies and human sera from a phase 1 clinical trial. The 2 studies are described in the sections below.

### Animal Studies

Crl:CD Sprague-Dawley rats were obtained from BioLASCO Taiwan Co Ltd, and studies were conducted in the Testing Facility for Biological Safety (TFBS Bioscience Inc). Immunizations of Sprague-Dawley rats were carried out as previously described [12]. In brief, rats (n = 10 for each dose group, 5 males and 5 females) were immunized 3 times at 2 weeks apart with 5, 25, or 50 µg of S-2P protein adjuvanted with 1500 µg of CpG 1018 and 750 µg of aluminum hydroxide. The sera of individual rats were harvested 2 weeks after the second immunization (day 29) and 2 weeks after the third immunization (day 43) and subjected to neutralization assay with pseudovirus expressing wild-type (WT) SARS-CoV-2 Wuhan-Hu-1 or Beta variant spike proteins.

All procedures in this study involving animals were conducted to avoid or minimize discomfort, distress, or pain to the animals and were carried out in compliance with the Animal Research: Reporting of In Vivo Experiments (ARRIVE) guidelines (https://arriveguidelines.org/). All animal work in the current study was reviewed and approved by the Institutional Animal Care and Use Committee with animal study protocol approval number TFBS2020–010.

### Clinical Trial

Forty-five human subjects (28 males and 17 females) aged 20–49 years (mean age, 33.8 years) were enrolled in a prospective, open-label, single-center dose-escalation phase 1 study with 3 subphases, each of which had 15 participants. The 3 different dose levels employed in this clinical trial were low-dose (5 µg), medium-dose (15 µg), and high-dose (25 µg) S-2P protein adjuvanted with 750 µg of CpG 1018 and 375 µg of aluminum hydroxide for phases 1a, 1b, and 1c, respectively. The vaccination schedule consisted of 2 doses, administered by intramuscular injection of 0.5 mL in the deltoid region of the nondominant arm, preferably 28 days apart, on day 1 and day 29. On day 57 (4 weeks after the second administration), serum samples were taken for neutralization assays with pseudoviruses expressing the spike protein of WT, D614G, Alpha, and Beta variants as outlined below. The trial was initiated in September 2020 and interim safety and immunogenicity results were published in June 2021 [13]. This phase 1 trial has been registered at ClinicalTrials.gov (NCT04487210).

### Pseudovirus Neutralization Assay

Lentivirus expressing the SARS-CoV-2 spike proteins of the Wuhan-Hu-1 WT strain was constructed, and the neutralization assay was performed as previously described [12]. Lentiviruses expressing D614G, Alpha, and Beta variant spike proteins were constructed in the same manner but with the WT spike protein sequence replaced with the respective variant sequences.

### Statistical Analysis

**Figure 1.**
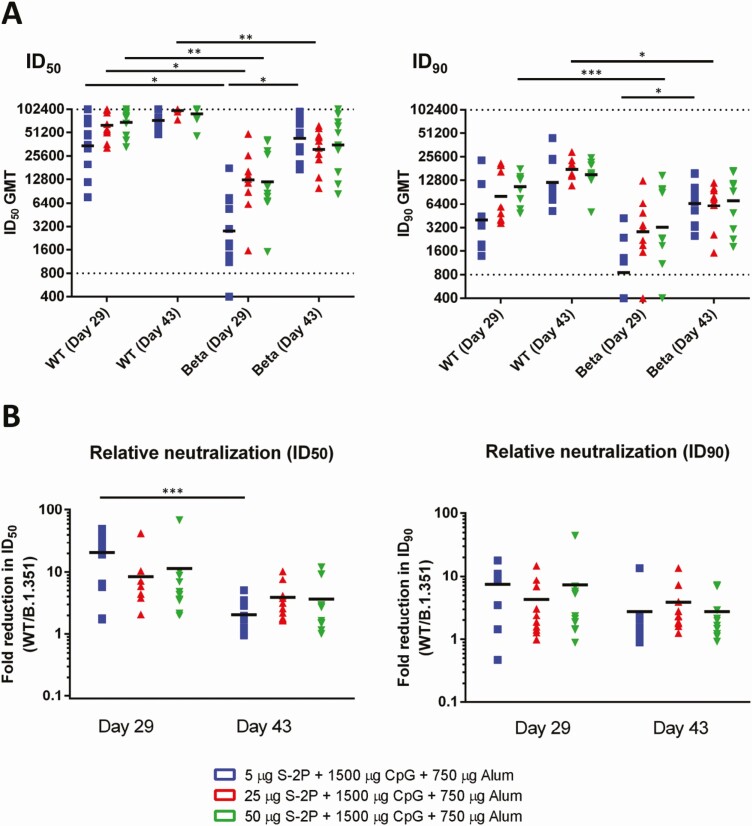
Neutralization of severe acute respiratory syndrome coronavirus 2 (SARS-CoV-2) pseudovirus bearing wild-type (WT) or Beta variant spike proteins by antisera of rats vaccinated with adjuvanted S-2P. Rats (n = 10 for each dose group) were immunized 3 times at 2 weeks apart with the indicated amounts of adjuvanted S-2P. *A*, The antisera were harvested 2 weeks after the second immunization (day 29) and 2 weeks after the third immunization (day 43) and subjected to neutralization assay with pseudovirus expressing WT or Beta variant SARS-CoV-2 spike protein to determine the 50% inhibition dilution (ID_50_) and 90% inhibition dilution (ID_90_) titers of neutralizing antibodies. *B*, Fold reductions in neutralizing titers (ID_50_ and ID_90_) were calculated by dividing WT neutralizing titers by Beta neutralizing titers. Results are presented in (*A*) as horizontal bars representing geometric mean titers (GMTs) with symbols representing individual titers, and (*B*) as horizontal bars representing means and symbols representing individual titer ratios. Dotted lines indicate the lower and upper limits of detection of the assay. Kruskal-Wallis with Dunnett multiple comparisons test was performed and statistical significance of neutralizing titers between day 29 and day 43 of the Beta variant and WT at different dose levels. *P < .05, **P < .01, ***P < .001.

**Figure 2.**
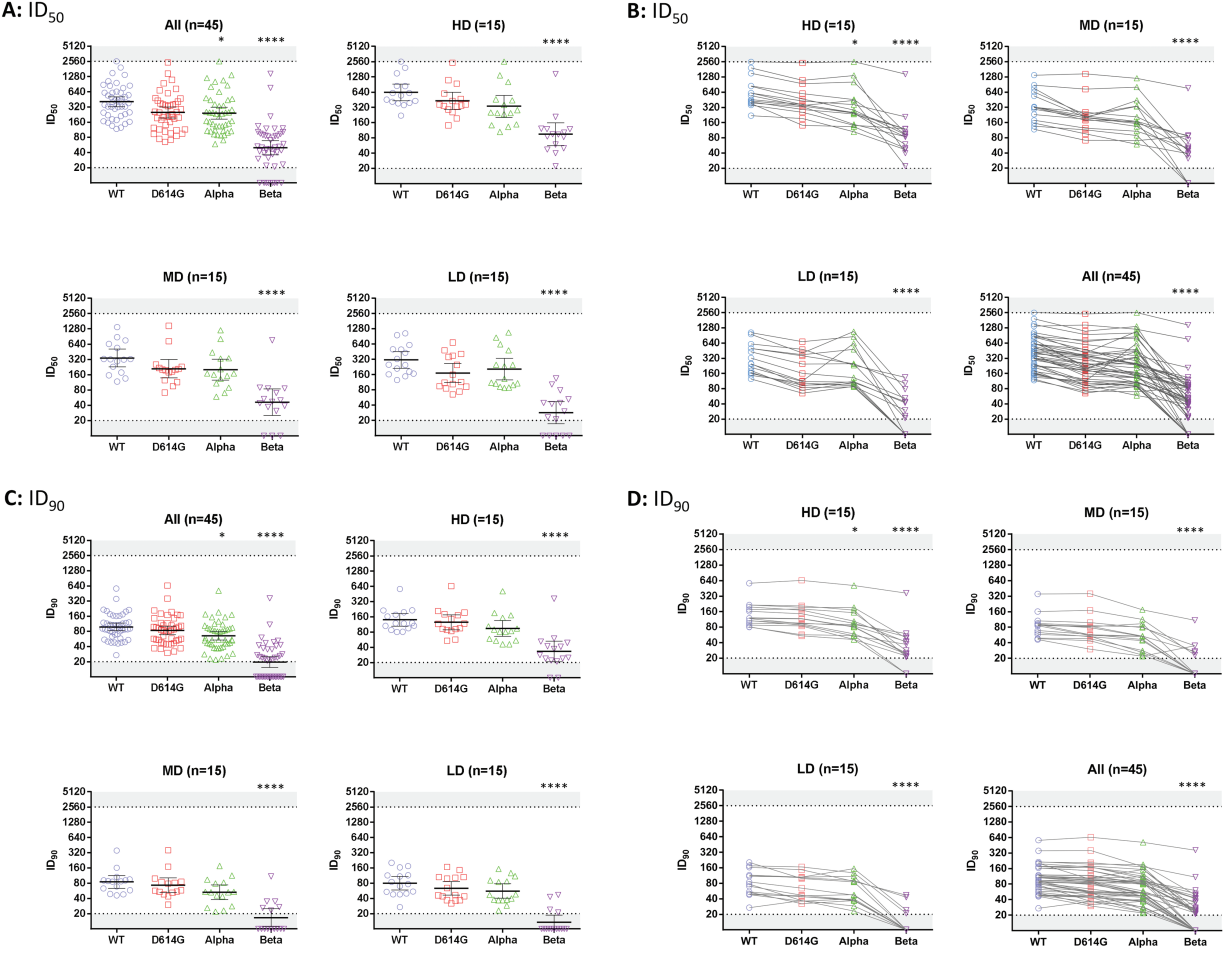
Neutralization of severe acute respiratory syndrome coronavirus 2 (SARS-CoV-2) pseudoviruses with wild-type (WT) or variant spike proteins by antisera of clinical trial subjects vaccinated with different doses of MVC-COV1901. Serum samples from subjects of the phase 1 clinical trial of MVC-COV1901 were collected 4 weeks after the second immunization (56 days from the first immunization). 50% inhibition dilution (ID_50_) (*A* and *B*) and 90% inhibition dilution (ID_90_) (*C* and *D*) neutralizing titers for low dose (LD), medium dose (MD), high dose (HD), and all dose groups were measured with pseudovirus neutralization assays. *A* and *C*, Geometric mean titers are represented by the horizontal bars with error bars representing 95% confidence interval and individual titer represented by symbols. *B* and *D*, Results are represented here with each symbol representing individual neutralizing titer and lines connecting neutralizing titer of the WT, D614G, Alpha, and Beta pseudovirus for each serum sample. Dotted lines and shaded areas indicate the lower and upper limits of detection of the assay. Kruskal-Wallis with Dunnett multiple comparisons test was performed to calculate the statistical significance of neutralizing titers between variants relative to WT. *P < .05, ****P < .0001.

No statistical methods were used to predetermine sample size used in the phase 1 clinical study and rat toxicology study. Values equivalent to or below the limit of detection were tabulated and calculated as half of the limit of detection (400 for Figure 1A and 1B; 10 for Figure 2). Prism 6.01 software (GraphPad) was used for statistical analysis. Shapiro-Wilk test for normality and Levene test for equal variances were performed, and the data did not meet criteria for parametric tests. As a result, Kruskal-Wallis with Dunnett test for multiple comparisons was used for statistical tests to compare results between WT and variants and as indicated in the figure legends. 

## RESULTS

### MVC-COV1901–Induced Antibodies in Rats Retain Effective Neutralization Ability Against the Beta Variant

We previously found that MVC-COV1901 induced robust neutralizing antibody responses against WT virus in rats during the course of toxicology studies. To investigate the neutralizing ability of MVC-COV1901–induced antibodies against VoCs, rat antisera collected from the toxicology study were subjected to neutralization assay with pseudovirus expressing SARS-CoV-2 WT or Beta variant spike protein. At days 29 and 43, the antibodies were able to neutralize the Beta variant (Figure 1), although the neutralizing titers were reduced. Notably, sera sampled 2 weeks after the third immunization (day 43) had higher geometric mean titers (GMTs) than sera sampled 2 weeks after the second immunization (day 29). The effect is especially pronounced in the low-dose group (5 µg), where Dilution required to reach 50% virus inhibition (ID_50_) and Dilution required to reach 90% virus inhibition (ID_90_) GMTs against Beta variant increased significantly (*P* = .015) from 2808 to 42 967, and from 850 to 6559 from day 29 to day 43, respectively. By day 43, all dose groups achieved similar levels of GMTs against the Beta variant at ID_50_ of 42 967, 31 010, and 35 484 for the 5-, 25-, and 50-µg doses, respectively, and ID_90_ of 6559, 6100, and 7109 for the 5-, 25-, and 50-µg doses, respectively (Figure 1A). The mean levels of fold reduction in ID_50_ of Beta variant relative to WT at day 29 were 20.5-, 8.47-, and 11.42-fold for the 5-, 25-, and 50-µg doses, respectively; at day 43 there were decreases of 2.0-, 3.9-, and 3.6-fold for the 5-, 25-, and 50-µg doses, respectively (Figure 1B).

Overall, we have shown that antibodies elicited by high-dose S-2P/adjuvant immunization in rats could effectively neutralize the Beta variant. Human antisera from vaccination with MVC-COV1901 neutralized the D614G and Alpha variants, but neutralization was diminished with the Beta variant.

**Figure 3.**
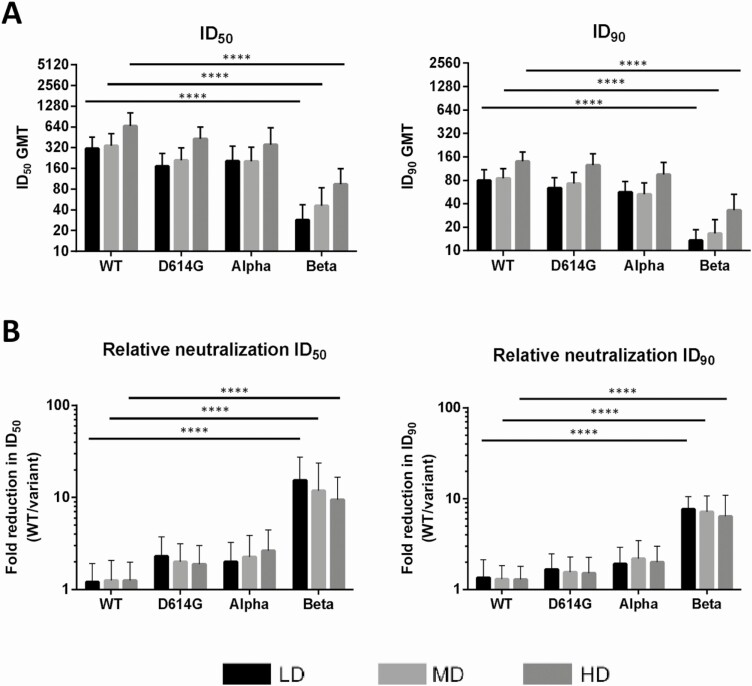
Neutralization of variant pseudoviruses by human antisera across different doses and fold reduction in the neutralization of D614G, Alpha, and Beta variants relative to wild-type (wt). *A*, 50% inhibition dilution (ID_50_) and 90% inhibition dilution (ID_90_) neutralizing titers of each dose group were plotted for the WT and variants with data from Figure 2. Bars and error bars represent geometric mean titers (GMTs) and 95% confidence intervals, respectively. *B*, Fold reduction in neutralization vs the WT was calculated by dividing the average ID_50_ and ID_90_ titers of WT by ID_50_ and ID_90_ titers of each variant. Results are plotted as bars and error bars indicating mean and standard deviation, respectively. Kruskal-Wallis with Dunnett multiple comparisons test was used to compare neutralizing titers and ratios of variants relative to WT at high-dose (HD), medium-dose (MD), and low-dose (LD) levels. ****P < .0001.

After confirming that antibodies induced by MVC-COV1901 in rats could neutralize the Beta VoC, pseudovirus neutralization assays were conducted with human serum samples taken from our phase 1 clinical trial drawn at 4 weeks after the second immunization with low-dose, medium-dose, or high-dose MVC-COV1901. Figure 2 presents the data from pseudovirus neutralization assays of human sera with the panel of pseudovirus expressing spikes of WT, D614G, Alpha, and Beta variants. In all dose levels, neutralizing titers were reduced in all variants compared to WT, with the Alpha variant having 1.7-fold (*P* = .0306) and 1.5-fold (*P* = .0223) decrease in GMT for ID_50_ and ID_90_, respectively. The Beta variant resulted in a significant (*P* < .0001) reduction in titers compared with WT, with the ID_50_ GMTs of low, medium, and high dose decreased from 311, 342, and 663 to 28, 46, and 94, respectively (Figure 2A), and ID_90_ GMTs of low, medium, and high dose decreased from 80, 87, and 119 to 13, 17, and 33, respectively (Figure 2C). Neutralizing titers against the Beta variant were increased at a higher dose of antigen (Figure 3A). Compared to WT, the Beta variant resulted in a 9.5- to 15.4-fold reduction of neutralizing titers for ID_50_ and 5.5- to 6.6-fold reduction in neutralizing titers for ID_90_ (Figure 3B). It is noted that although the neutralizing titers increased from low dose to high dose as shown in Figure 3A (eg, ID_50_ GMTs of 311, 342, and 633 against WT and 28, 46, and 94 against the Beta variant in low-, medium-, and high-dose levels, respectively), the differences between GMTs of each dose level were not found to be statistically significant (*P* > .05).

## Discussion

In this study, we showed that in humans, 2 injections of a subunit vaccine consisting of the prefusion spike protein (S-2P) adjuvanted with CpG 1018 and aluminum hydroxide were effective in inducing potent neutralization activity against pseudovirus expressing WT, D614G, and Alpha variant spike proteins, but to a lesser extent than the Beta variant. Our study showed that the neutralizing titers of phase 1 human antisera against the Beta variant, compared to WT, had 5.5- to 6.6-fold (ID_90_) reduction, which is comparable to results from studies using Moderna and Pfizer’s vaccinee sera as reported by Wang et al and others [6, 14, 15]. Furthermore, their data are also consistent with our findings, which show slight changes of neutralizing titers against the pseudoviruses of D614G or Alpha variant. In our case, with a fixed dose of CpG 1018 and aluminum hydroxide, the data showed that the absolute neutralizing titers against the Beta variant correlate with the amount of S-2P antigen in the formulation (Figure 3A).

The neutralizing antibodies elicited by MVC-COV1901’s spike protein trimers are polyclonal, as multiple antigenic epitopes could be identified on the protein. The monoclonal antibody (mAb) activities abolished by any mutated epitope could theoretically be compensated for when titers of other neutralizing monoclonal antibodies increase. Amanat et al profiled polyclonal antibodies induced by a SARS-CoV-2 spike mRNA vaccine [16]. They demonstrated the co-dominance of mAbs targeting the N-terminal domain (NTD) and RBD. The mAbs targeting RBD showed smaller abolishment of neutralizing activities against viral variants carrying E484K than mAbs to NTD. The competing mAbs bind differentially to variants, suggesting the protective importance of the otherwise redundant mAbs against the VoCs [17]. It is unclear if the proportions of neutralizing mAbs targeting NTD and RBD would change as the dose escalation was conducted in our first-in-human study, but the overall higher neutralizing antibody titer among the high-dose antigen group means a higher dose of effective mAbs against the viral variant in question (Figures 1 and 2). Our findings suggest that the antigen amount with a fixed adjuvant formulation correlates with the neutralizing antibody titers against VoCs. The study results by Garcia-Beltran et al using sera of 99 vaccinees of Pfizer and Moderna COVID-19 vaccines demonstrated that after the second dose of both vaccines, the neutralizing antibody titers against the pseudoviruses of VoCs increase significantly, especially against the Beta variant [5]. Other studies using serum samples from Moderna, Pfizer, and Johnson & Johnson vaccinees have also found that while neutralizing antibody titers were reduced, especially against the Beta, Gamma, and Delta variants, they remained effective in their neutralization capabilities [18–20]. Furthermore, it has been reported that neutralizing antibodies against WT and variants induced by mRNA-1273 vaccine were able to persist at lower level for at least 6 months after 2 vaccinations, and administration of a booster shot of either WT or variant spike mRNA 6 months after the second vaccination could boost immunogenicity against variants [9, 21]. The above findings lead to our thinking that it might be a viable option to combat the VoCs by increasing the prototype antigen, the adjuvant, or the number of shots, without redesigning the antigen. In the rat study as presented in Figure 1, by using a 3-dose regimen, we were able to induce similar levels of neutralizing titer across 3-dose groups. Compared to 2 weeks after the second dose (day 29), the GMTs of the medium-dose and high-dose groups against Beta did not increase significantly by 2 weeks after the third dose (day 43), indicating that the GMT levels have plateaued by day 43, while the ID_50_ GMT in the low-dose group had a 15-fold (*P* = .0148) increase (Figure 1A). Given that the 3-dose regimen resulted in high immunogenicity against the variant, these results could be extrapolated to mean that in humans an extra immunization could be a strategy to increase immunity against the VoCs. At this moment, only a handful of real-world vaccine efficacy data against the variants have been published or released. Similar to the immunogenicity data, the Alpha variant was shown to be less resistant to vaccine-induced immunity, with ChAdOx1 nCoV-19 having efficacies of 70.4% and 81.5% against the Alpha variant and non-Alpha strains, and NVX-CoV2373 having efficacies of 86.3% and 96.4% against the Alpha variant and WT, respectively [22, 23]. Against the Beta variant, ChAdOx1 nCoV-19 performed poorly with an efficacy of only 21.9% against mild to moderate disease due to the Beta variant, whereas it fared better against the Delta variant with an efficacy of 67% [24]. The more recent studies focusing on the circulating Delta variant showed that although the efficacy has decreased as with other variants, the vaccines still remained highly effective against hospitalization, with the BNT162b2 and ChAdOx1 nCoV-19 vaccines offering 96% and 92% effectiveness against hospitalization after 2 doses [25, 26].

Our data do not allow us to conclude whether increasing the amount of antigen given or increasing the number of vaccine doses given is more impactful in eliciting neutralizing antibodies against variants. However, since our data suggest that the amount of antigen correlates with neutralization of VoCs, further investigation of the strategy of boosting neutralizing antibody titer to VoCs with higher amount of antigen or adjuvant, or higher number of shots, seems warranted.

Decision making to initiate development of new COVID-19 vaccines to cope with VoCs is inherently challenging due to the following unknowns: the clinical significance of the emerging strain, the geographical distribution where the strain will be predominant, and how they will mutate further. The US Food and Drug Administration has published guidance for the industry to change the antigen based on the VoCs to gain regulatory approval using the data accrued from the earlier development stages [27]. Nevertheless, the rollout of variant-specific vaccine that targets a geographical area where the strain is dominant can compromise a vaccination program’s feasibility and timeliness, particularly in low- and middle-income settings. Furthermore, it is unknown if the phenomenon of “original antigenic sin,” in which the development of immunity is shaped by the version of pathogen first encountered, would cause a problem for COVID-19, as a redesigned antigen might preferentially boost the antibodies elicited by the original antigen [28]. Thus, it is desirable to have a COVID-19 vaccine that can cover the emerging variants for a given period before the accumulated mutations lead to a clinically significant abolishment of the neutralizing capacity generated by the vaccine.

Among the emerging VoCs, the Beta variant first reported in the Eastern Cape of South Africa is more problematic. An in vitro study has demonstrated immune escape of the variant from convalescent plasma collected from individuals infected by earlier variants. An ID_50_ attenuation of neutralizing titers of up to 200-fold was reported [29]. The findings imply that individuals may be reinfected by the Beta variant even after a previous episode of SARS-CoV-2 infection that generates neutralizing titer [30]. It raises the question of whether COVID-19 vaccines that were designed based on the original virus could yield protection against the variant at the public health level. This study investigates how the MVC-COV1901 performs in vitro against the VoCs to evaluate if MVC-COV1901 can yield clinical protection and inform further development strategy.

A limitation of our study is that the sera were taken 4 weeks after the second shot of MVC-COV1901 when immunity has peaked and only just started to wane. Therefore, we could not evaluate the impact of waning immunity on the neutralizing capacity against the variants. The study cannot evaluate the role of T-cell responses elicited by the vaccine as it was reported that the T-cell responses to immunization may confer heterotypic coverage and are less affected by VoCs [31]. COVID-19 vaccines in phase 3 clinical trials in South Africa when the Beta variant became predominant showed considerably high protection against severe clinical endpoints, while overall efficacy is lower than at sites outside of South Africa [32]. Since there are no correlates of protection being published for emerging variants, it warrants further study on how the reduced neutralizing titers will translate to clinical endpoints. We are currently conducting a randomized, double-blind, placebo-controlled phase 2 study for safety and immunogenicity of MVC-COV1901 with >3800 subjects (NCT04695652). The samples from the phase 2 trial would allow us to further investigate the effects of variants on neutralizing titers by using a larger set of samples and newer variants.

## Notes

***Acknowledgments.*** We are grateful to TFBS Biosciences for toxicology studies and sampling involving rats. We thank Hao-Yuan Cheng, I-Chen Tai, and Erh-Fang Hsieh of the clinical team for planning and execution of the phase 1 clinical trial. We also thank John D. Campbell and Paula Traquina of Dynavax Technologies for reviewing and providing helpful inputs toward the manuscript and experimental design. We thank Michael Kornacker for reviewing and proofing of the manuscript. The datasets generated during and analyzed during the current study are available from the corresponding author upon reasonable request.

***Author contributions.*** C.-E. L., T.-Y. K., M.-Y. L., and C. C. coordinated the projects. T.-Y. K., W.-C. L., J. C., and Y.-C. C. produced S-2P antigen and pseudoviruses used in the study. C.-E. L., T.-Y. K., Y.-J. L., M.-Y. L., and Y.-C. C. designed the study and experiments. Y.-J. L., M.-Y. L., L. T.-C. L., and Y.-C. C. performed and analyzed the experiments. C.-E. L., M.-Y. L., and L. T.-C. L. drafted the manuscript. All authors have reviewed and approved the final version of the manuscript.

***Potential conflicts of interest.*** C.-E. L., T.-Y. K., Y.-J. L., W.-C. L., M.-Y. L., L. T.-C. L., J. C., and C. C. are employees of Medigen Vaccine Biologics Corporation and may hold stock or stock options. T.-Y. K., Y.-J. L., M.-Y. L., and C. C. are co-inventors of patents pending US63/040,696, TW110122289, PCT/CN021/100826, and US17/351363. Employees of Medigen Vaccine Biologics Corporation reported grants from the Taiwan Centers for Disease Control, Ministry of Health and Welfare for the phase 1 clinical trial. Y.-C. C. reports no potential conflicts of interest.
